# Clinical Characteristics of Children With Acute Tubulointerstitial Nephritis: A Single-Center Experience

**DOI:** 10.7759/cureus.36379

**Published:** 2023-03-19

**Authors:** Emre Leventoğlu, Bahriye Uzun Kenan, Bahar Büyükkaragöz, Sevcan A Bakkaloğlu

**Affiliations:** 1 Pediatric Nephrology, Faculty of Medicine, Gazi University, Ankara, TUR

**Keywords:** kidney injury, steroid therapy, kidney biopsy, acute tubulointerstitial nephritis, children

## Abstract

Objective: Acute tubulointerstitial nephritis (ATIN) is an infiltration of the kidney interstitium with inflammatory cells. Medications are most frequently blamed for the etiology. Patients may present with non-specific signs and symptoms. Therefore, the diagnosis of ATIN is often delayed. In this study, clinical characteristics, treatment protocols, and outcomes of children diagnosed with ATIN were presented.

Methods: This is a retrospective study based on the data of 18 patients diagnosed with ATIN between 2017 and 2022 at Gazi University. Patients were divided into two groups: steroid-treated (n=13) and non-steroid-treated (n=5). Clinical features and laboratory evaluations were compared between the groups.

Results: The mean age of the patients was 14.4±2.6 years, and the great majority were girls (88.9%, n=16). ATIN was mostly medication-related (n=17, 94.4%). Steroids were started in one-third of patients using non-steroidal anti-inflammatory drugs. Steroids were started in 45.4% of the patients with eosinophilia, 75% of those with pyuria, 66.6% of those with hematuria, and half of the patients with increased kidney echogenicity. The kidney functions returned to normal ranges in all patients. In steroid-treated patients, although recovery times for serum creatinine were longer (7.2±2.5 vs. 71.2±100.7 days), blood eosinophil count reached normal values more rapidly (5.4±2.3 vs. 3.1±1.0 days).

Conclusion: ATIN can be associated with diverse clinical presentations. The first and most important step of treatment is to discontinue the medication responsible for the etiology. Steroid treatment improves eosinophilia more rapidly. However, randomized controlled studies are needed to determine further treatment steps and establish a more definite treatment protocol.

## Introduction

Acute tubulointerstitial nephritis (ATIN) is an infiltration of the renal interstitium with inflammatory cells, including neutrophils, monocytes, lymphocytes, and eosinophils [1]. It is observed at a rate of 3-7% in kidney biopsies in children [2]. Among the causes of ATIN, medications (non-steroidal anti-inflammatory drugs (NSAIDs), beta-lactam antibiotics, and proton pump inhibitors (PPIs)) are most frequently blamed. This is followed by infections, immune-mediated diseases, tubulointerstitial nephritis and uveitis (TINU) syndrome, granulomatous diseases, and genetic causes. In a significant group of patients, no causative agent could be detected [1].

In ATIN of any cause, patients may present with non-specific signs and symptoms of acute kidney dysfunction. These include the acute or subacute onset of nausea, vomiting, and malaise [3]. In drug-induced ATIN, extrarenal manifestations of hypersensitivity, such as fever, skin rash, and eosinophilia, are relatively common. However, many patients are asymptomatic [4]. Therefore, since patients usually present with non-specific symptoms and findings, the diagnosis of ATIN is often delayed. While the majority of patients recover spontaneously, a severe clinical picture that may rarely progress to kidney failure may be observed [5].

In this study, clinical characteristics, treatment protocols, and outcomes of pediatric patients diagnosed with ATIN who were followed up in a single center were presented.

## Materials and methods

Study design

This is a retrospective study based on data collected from children and adolescents diagnosed with ATIN between 2017 and 2022 at the Department of Pediatric Nephrology, Gazi University.

All data were retrospectively obtained from the electronic medical record system. Demographic characteristics, complaints at presentation, physical examination findings, and possible etiological factors (including the presence of medication use, previous infections, or chronic diseases) were evaluated. Laboratory values, including complete blood count (white blood cell, neutrophil, lymphocyte, and eosinophil counts); serum biochemistry, including serum creatinine and albumin levels; dipstick examination; and urine microscopy findings (urine density, proteinuria, hematuria or pyuria) were noted at admission and at the last follow-up. Urinary system ultrasonography and kidney biopsy findings (if available) were recorded.

Especially in drug-related ATIN, spontaneous recovery may be achieved with early discontinuation of the medication [1]. However, persistence of kidney dysfunction (persistence of elevated serum creatinine, persistent proteinuria, and/or hematuria) after discontinuation of the related medication is a major indication for commencement on steroids [6]. In our study, patients who were started on steroids because of persistently elevated creatinine, proteinuria, and/or hematuria were retrospectively evaluated, and these patients were grouped in a separate group. Steroid dose and duration were noted in the group receiving steroid therapy. Demographic and laboratory values and differences in terms of rates and durations of recovery were compared in these two groups. Kidney clinical improvement was defined as a decrease in serum creatinine to its basal value and improvement of proteinuria, hematuria, and pyuria.

This study was approved by Gazi University Clinical Research Ethics Committee with the approval number 2022-1467.

Statistical analysis

In the presentation of descriptive statistics, the data obtained by measurement were expressed as mean ± standard deviation (SD) and categorical data as number (percentage). Cross-table analyses and Fisher's exact chi-square tests were used to compare the qualitative characteristics of the groups. The Shapiro-Wilk test was used to determine the normal distribution of numerical measurements in groups. Two groups were compared with the t-test in independent groups and Mann-Whitney U test for those who did not show normal distribution. IBM SPSS Statistics for Windows, Version 22.0 (Released 2013; IBM Corp., Armonk, New York, United States) was used for all statistical analyses. A significance level of p<0.05 was taken.

## Results

Eighteen patients were included in the study. The mean age of the study group was 14.4±2.6 years, and the great majority were girls (88.9%, n=16). The mean weight and height z-scores of the patients were within normal intervals for age (0.67±1.32 and 0.60±0.55, respectively).

The most common complaints at initial admission were nausea and vomiting (77.8%, n=14). These were followed by flank pain, fever, malaise, and weight loss. On physical examination, costovertebral angle (CVA) tenderness was present in 61.1% of the patients (n=11). None of the patients had uveitis. The etiologic evaluation revealed that half of the patients (50%, n=9) used NSAIDs before the onset of signs and symptoms. This was followed by beta-lactam antibiotics and PPIs. One (5.6%) patient was taking a medication containing the active substance mirtazapine, and only one (5.6%) patient revealed no prior medication exposure. There were no patients taking more than one medication at the same time or using an herbal-based product. The patient on mirtazapine had been taking the medication for about three months, and one of the patients on NSAIDs had been taking the medication for about 20 days. Except for these two patients, there was no history of chronic medication use. None of the patients had a history of infection or other chronic/systemic diseases.

The mean serum creatinine level and urinary protein excretion were 2.08±1.06 mg/dL and 9.0±4.6 mg/m^2^/h, respectively, whereas urine density was low (1005.8±4.4). No patient required kidney replacement therapy. Hypoalbuminemia was observed in 16.7% (n=3) of the patients, and eosinophilia was observed in 61.1% (n=11). In urine dipstick evaluation, 27.8% (n=5) of the patients had 2+ proteinuria. Moreover, leukocyturia was detected in 22.2% (n=4) of the cases and hematuria in 11.1% (n=2). Kidney parenchymal echogenicity was increased in 10 (55.6%) of the patients on ultrasonography. Five (27.7%) patients underwent kidney biopsy. Kidney biopsy showed interstitial infiltrates and interstitial edema consisting of mononuclear cells, mainly lymphocytes and eosinophils, which were diffuse in one patient (chronic mirtazapine-used patient) and focal in the other four patients.

The clinical and laboratory characteristics of the patients are shown in Table 1.

**Table 1:** The clinical and laboratory characteristics of the patients. BMI: body mass index; CVA: costovertebral angle; NSAID: non-steroidal anti-inflammatory drug; PPI: proton pump inhibitor; eGFR: estimated glomerular filtration rate; HPF: high power field.

	Mean ± SD	Min-Max	n (%)
Demographic features			
Age (years)	14.4±2.6	5-17	
Female			16 (88.9)
Anthropometric measurements			
Weight z-score	0.67±1.32	0.56-1.54	
Height z-score	0.60±0.55	0.22-1.43	
BMI z-score	1.14±0.77	0.78-1.55	
Complaints at presentation			
Nausea and vomiting			14 (77.8)
Flank pain			4 (22.2)
Malaise			3 (16.7)
Fever			4 (22.2)
Weight loss			3 (16.7)
Physical examination			
CVA tenderness			11 (61.1)
Evaluation of etiological agent			
NSAID			9 (50.0)
Beta-lactam antibiotics			5 (27.8)
PPI			2 (11.1)
Other (mirtazapine)			1 (5.6)
Unknown			1 (5.6)
Laboratory analysis			
Blood			
White blood cell (/µL)	8122.8±2240.4	3730-12800	
Neutrophil (/µL)	5263.1±2175.0	2180-9030	
Lymphocyte (/µL)	1932.8±543.4	1092-2730	
Eosinophil (/µL)	118.7±95.4	0-330	
Eosinophilia			11 (61.1)
Serum creatinine (mg/dL)	2.08±1.06	0.80-4.93	
eGFR (mL/min/1.73m^2^)	34.1±15.6	16.9-54.3	
Serum albumin (g/dL)	3.9±0.5	3.3-4.7	
Hypoalbuminemia			3 (16.7)
Urine			
Density	1005.8±4.4	1002-1017	
Hyposthenuria			15 (83.3)
Protein (dipstick)			
Negative			10 (55.6)
Trace			3 (16.7)
1+			0 (0)
2+			5 (27.8)
Leukocyte (/HPF)	13.9±12.1	0-63	
Pyuria			4 (22.2)
Erythrocyte (/HPF)	7.5±5.6	0-28	
Hematuria			2 (11.1)
Protein (mg/m^2^/h)	9.0±4.6	3.3-19.8	
Proteinuria (24 h)			17 (94.4)
Volume (mL/day)	1821.5±298.4	500-2500	
Kidney ultrasonography			
Increased echogenicity			10 (55.6)

All patients were hospitalized, and steroid therapy was started at 1 mg/kg/day (maximum 60 mg/day) doses in five (27.7%) patients with no decrease or further rise in serum creatinine levels during the follow-up. The mean duration of steroid use was 3.37±3.29 (0.5-9) months. Although the mean age of the patients who necessitated steroid therapy in addition to supportive measures seemed to be higher than those who used supportive treatment only, the difference was not statistically significant (p>0.05). The frequency of male sex was also higher in the steroid group (p>0.05). Height, weight, and body mass index (BMI) z-scores were numerically similar in both groups (p>0.05). Although not statistically significant, nausea and vomiting were more frequent in the steroid-free group (p=0.261). All other complaints, such as flank pain, malaise, fever, or weight loss, were more frequent in the steroid group (p>0.05, for all). A prior history of NSAID intake as an insulting agent was found to be the most common cause in both groups, but the rate was slightly higher in patients who needed to start steroid therapy (p=0.599). Steroids were not initiated in any of the patients who used beta-lactam antibiotics or PPIs (p=0.103 and p=0.352, respectively). In the steroid group, eosinophilia was present in all patients while hypoalbuminemia was not detected in anyone (p=0.036 and p=0.239, respectively). The majority of the patients with pyuria (75%) or hematuria (66.6%) necessitated steroid treatment, which was significantly higher than those without these findings (p=0.017 and p=0.016, respectively). Hyposthenuria was more frequent in the steroid-free group (p=0.099), whereas proteinuria (dipstick) was less frequent in that group (p=0.132). In addition, kidney echogenicity was increased in all patients in the steroid group (p=0.019), and steroid was used in all patients who underwent kidney biopsy (n=5, p<0.001). Clinical and laboratory values between the groups followed up with supportive treatment only and the groups in which steroids were added to the treatment are compared in Table 2.

**Table 2:** Clinical and laboratory values between the groups followed up with supportive treatment only and the group in which steroids were added to the treatment. BMI: body mass index; CVA: costovertebral angle; NSAID: non-steroidal anti-inflammatory drug; PPI: proton pump inhibitor; eGFR: estimated glomerular filtration rate; HPF: high power field.

	Supportive therapy (n=13)			Supportive + steroid therapy (n=5)			p value
	Mean ± SD	Min-Max	n (%)	Mean ± SD	Min-Max	n (%)	
Demographic features							
Age (years)	14.3±3.0	5-17		14.9 ± 1.2	14-17		0.562
Male			1 (7.7)			1 (20)	0.457
Anthropometric measurements							
Weight z-score	1.20±0.36	0.70-1.54		1.27±0.18	0.97-1.46		0.645
Height z-score	1.17±0.14	0.41-1.45		1.14±0.26	0.77-1.43		0.555
BMI z-score	1.23±0.19	0.99-1.33		1.31±0.36	1.12-1.45		0.479
Complaints at presentation							
Nausea and vomiting			11 (84.6)			3 (60)	0.261
Flank pain			2 (15.4)			2 (40)	0.299
Malaise			2 (15.4)			1 (20)	0.650
Fever			2 (15.4)			2 (40)	0.299
Weight loss			2 (15.4)			1 (20)	0.650
Physical examination							
CVA tenderness			8 (61.5)			3 (60)	0.952
Evaluation of etiological agent							
NSAID			6 (46.2)			3 (60)	0.599
Beta-lactam antibiotics			5 (38.5)			0 (0)	0.103
PPI			2 (15.4)			0 (0)	0.352
Other (mirtazapine)			0 (0)			1 (20)	0.097
Unknown			0 (0)			1 (20)	0.097
Laboratory analysis							
Blood							
White blood cell (/µL)	7929.5±2320.6	3730-12800		8625.6±2177.3	6404-11400		0.375
Neutrophil (/µL)	5087.6±2153.6	2180-9030		5720.0±2414.8	3200-8600		0.361
Lymphocyte (/µL)	1994.7±501.0	1092-2730		1772.6±677.1	1180-2500		0.224
Eosinophil (/µL)	85.9±76.1	0-210		204.0±93.6	100-330		0.014
Eosinophilia			6 (46.2)			5 (100)	0.036
Serum creatinine (mg/dL)	2.16±1.24	0.80-4.93		1.87±0.29	1.52-2.16		0.492
eGFR (mL/min/1.73m^2^)	34.4±12.7	10.3-54.3		36.4±19.9	9.3-47.8		0.379
Serum albumin (g/dL)	3.94±0.54	3.3-4.7		4.14±0.40	3.7-4.5		0.932
Hypoalbuminemia			3 (23.1)			0 (0)	0.239
Urine							
Density	1005.2±3.6	1002-1015		1007.4±6.3	1002-1017		0.222
Hyposthenuria			12 (92.3)			3 (60)	0.099
Protein (dipstick)							
Negative			8 (61.5)			2 (40)	0.132
Trace			3 (23.1)			0 (0)	
1+			0 (0)			0 (0)	
2+			2 (15.4)			3 (60)	
Leukocyte (/HPF)	5.9±3.3	0-10		23.6±4.4	4-63		0.014
Pyuria			1 (7.7)			3 (60)	0.017
Erythrocyte (/HPF)	6.3±5.9	0-13		8.7±7.1	0-28		0.886
Hematuria			1 (7.7)			2 (40)	0.016
Protein (mg/m^2^/h)	6.5 ±2.2	3.3-11.2		15.5±2.1	13.9-19.8		0.000
Proteinuria (24 h)			12 (92.3)			5 (100)	0.523
Volume (mL/day)	1744.4±259.6	1100-2300		1637.3±376.7	300 -2500		0.496
Kidney ultrasonography							
Increased echogenicity			5 (38.5)			5 (100)	0.019

At the end of the follow-up period of mean 31.2±19.1 (5-60) months, kidney functions were normalized in all patients. Aside from serum creatinine, eosinophil count, urinary protein excretion, pyuria, and hematuria rates decreased, whereas urine density increased in all patients. On the other hand, the time for serum creatinine to reach basal value and for 24-h urinary protein excretion level to regress to normal range were longer in the steroid-treated patients compared to patients on supportive treatment (p=0.028 and p=0.040, respectively). In the steroid-treated group, two patients had a history of chronic medication use, and one of these two patients had diffuse rather than focal inflammation on kidney biopsy, unlike the other patients (p<0.001). They had the longest time for mean serum creatinine to return to baseline 165.0±106.0 (90-240) days compared to the rest of the patients (p<0.001). The blood eosinophil count reached normal values more rapidly in the steroid users (p=0.049). Although not statistically significant, urine density also normalized more quickly in patients treated with steroids (p>0.05). The time required for the normalization of the laboratory parameters is shown in Table 3.

**Table 3:** The time required for the normalization of the laboratory parameters.

	Supportive therapy		Supportive + steroid therapy		p value
	Mean ± SD	Min-Max	Mean ± SD	Min-Max	
For serum creatinine (days)	7.2±2.5	4-15	71.2±100.7	8-240	0.028
For eosinophilia (days)	5.4±2.3	2-8	3.1±1.0	1-4	0.049
For 24-h proteinuria (days)	24.5±14.1	7-31	51.4±72.4	4-195	0.040
For urine density (days)	4.6±2.1	2-7	3.2±2.9	1-10	0.245

Two of the patients were re-exposed to the causative agents (PPI and beta-lactam antibiotics) a few months after the diagnosis of ATIN, but no clinical or laboratory abnormalities were detected in these patients.

## Discussion

In this study, all ATIN cases were drug-induced, except for a patient with an undetermined etiology. Corticosteroid therapy was used in half of the patients with flank pain and fever. Although steroids were not initiated in any of the patients who were notified to have beta-lactam antibiotics or PPI use in the etiology, one-third of patients with a history of NSAID use required the treatment. We started steroids in about half of the patients (45.4%) with eosinophilia and in more than half of the patients with pyuria (75%) or hematuria (66.6%). Besides, it was used in half of the patients with increased kidney parenchymal echogenicity and in all patients who underwent kidney biopsy (all of whom had interstitial inflammation). At the end of the follow-up period, kidney functions returned to normal ranges in all patients irrespective of steroid use. However, recovery times for serum creatinine and proteinuria were significantly longer in steroid-treated patients. The blood eosinophil count reached normal values faster in the steroid users.

The results regarding sex distribution in ATIN are quite variable. In one single-center study, 57.9% of pediatric patients diagnosed with ATIN were girls, and in another single-center study, the proportion of girls was 90% [4,7]. In our study, 88.9% of the study group consisted of girls. There is no study in the literature for ATIN and sex predominance; however, a study has shown that drug allergy is more common in girls [8]. Since medication exposure was frequently found in the etiology of ATIN in our study and an allergic component is thought to be present in ATIN, a higher frequency of female sex may be an expected finding [1].

ATIN has a wide clinical spectrum ranging from acute kidney injury, which may improve spontaneously by removal of the etiological factor, to very severe kidney involvement that requires dialysis [5]. In 72.2% of our patients, elimination of the possible etiological factors was sufficient to normalize acute kidney injury findings spontaneously over time. In the remaining patients, steroids were started in addition to supportive therapy.

Symptoms and signs of ATIN may be nonspecific, but uremic symptoms may occur if kidney failure develops [9]. The absence of the classic triad of fever, eosinophilia, and allergic rash does not exclude ATIN. Minimal proteinuria is frequently found in patients, but nephrotic level proteinuria may develop in rare cases. A routine urine analysis may reveal the presence of white or red blood cells [6]. Eosinophiluria may also be demonstrated by Hansel’s stain [10]. However, assessment for eosinophiluria was lacking in our study due to technical issues. In our patients, non-specific symptoms such as weakness, fever, weight loss, nausea, and vomiting were the most common complaints. Since none of our patients had an allergic rash, no one fulfilled the classic triad of ATIN. Although some of our patients had some degrees of proteinuria, none of them showed nephrotic level proteinuria. Some of our patients also had pyuria and hematuria.

The diagnosis of ATIN can be confirmed by kidney biopsy due to the presence of focal or diffuse interstitial infiltrates consisting predominantly of mononuclear cells, including lymphocytes and eosinophils, and interstitial edema. A biopsy is undertaken when the diagnosis is unclear or when the patient does not improve clinically following discontinuation of the medication suspected as the cause of AIN and kidney failure [11]. In our patients, interstitial infiltrates accompanied by eosinophils and interstitial edema were present in all patients who underwent biopsy. However, a mild degree of tubulointerstitial fibrosis was observed in the patient with chronic use of the offending medication, mirtazapine.

Treatment is based on the clinician's previous experience. Supportive therapies such as close monitoring of intravascular volume and maintenance of electrolyte balance are essential. Kidney function may improve with treatment of the underlying cause. Especially in drug-related ATIN, spontaneous recovery may be achieved with early discontinuation of the medication [1]. However, persistence of kidney dysfunction after discontinuation of the related medication is a major indication for the initiation of steroids [10]. In our study, supportive measures were applied in the majority of our patients (72.2%), and kidney functions normalized in a short time with the elimination of the possible etiological agent. However, steroid therapy was added to the supportive treatment for patients whose kidney dysfunction persisted after discontinuation of the medication implicated in the etiology.

There is no consensus in terms of corticosteroid dose and duration of use [11]. In a study by Gonzalez et al., patients diagnosed with drug-related ATIN were classified according to the presence of steroid treatment or not, and final serum creatinine was found to be significantly lower in the group receiving steroids compared to those without. Moreover, almost half of the non-steroid group had to be admitted for chronic dialysis sessions. In that study, it was also shown that when there was a delay in the initiation of steroid treatment, kidney functions did not fully recover [12]. In another study, patients who received prednisolone 2 mg/kg/day (maximum 60 mg/day) for one month followed by a gradual tapering in medication doses were compared with patients who were on only supportive treatment, and despite a rapid decrease in serum creatinine in the steroid users, no significant difference was found in serum creatinine levels at the end of treatment [13]. In our patients, the starting dose was 1 mg/kg/day in the steroid-treated group. Although urine density and serum eosinophil levels returned to normal ranges more rapidly in the steroid users, proteinuria and high serum creatinine persisted for a much longer time. We attribute the delayed recovery of markers associated with clinical improvement in the steroid-treated group to the presence of chronic medication use in two patients in this group. In summary, we believe that early discontinuation of the medication implicated in the etiology and/or early steroid treatment may lead to a decrease in inflammation and thereby the risk of fibrosis, and it may induce rapid recovery.

Most patients have a complete renal recovery in the long term, like our patients. Chronic kidney disease rarely develops. Progression into a chronic process is usually dependent on the underlying cause. Exposure to the triggering medication for more than one month, delay in removal of the triggering agent (like in our patient with mirtazapine use), especially drug-associated ATIN, systemic inflammatory state, genetic predisposition, prolonged acute kidney injury, intense neutrophil infiltration on biopsy, diffuse inflammation or severe fibrosis, and the presence of interstitial granuloma are indicators of poor prognosis. A few numbers of subjects and inclusion of only patients with medication exposure in the ATIN etiology can be considered as the main limitations of this study. Nevertheless, although previous studies report that initial clinical symptoms and laboratory tests (serum creatinine, urine analysis, and amount of proteinuria) do not have distinguishing features in terms of renal prognosis [1], our study showed that patients with echogenic kidneys, eosinophilia, and an active urine sediment at the onset of the disease were associated with significantly higher percentage of steroid use in the course of the disease. Therefore, we believe that careful clinical assessment and early initiation of steroids in the presence of indicators suggestive of a more severe disease will have a beneficial effect on the long-term prognosis of patients with ATIN.

## Conclusions

ATIN is a major cause of acute kidney injury in children. It can be associated with diverse clinical presentations that range from an asymptomatic, spontaneously recovering clinical state to a very severe clinical course that progresses into kidney failure. The first and most important step of treatment is elimination of the etiological condition. Another important step in treatment is the early initiation of steroid therapy, which can trigger rapid healing by reducing inflammation and thus the risk of fibrosis. However, randomized controlled studies are needed to determine further treatment steps and establish a more definitive treatment protocol.
